# Comparison of Gasserian ganglion conventional radiofrequency ablation and peripheral nerve pulsed radiofrequency in trigeminal neuralgia: a retrospective cohort study

**DOI:** 10.22514/jofph.2025.063

**Published:** 2026-01-12

**Authors:** Mesut Bakır, Gülçin Gazioğlu Türkyılmaz, Nurettin Teker, Kaan Yavuz, Bedri İlcan, Şebnem Rumeli

**Affiliations:** ^1^Division of Pain Medicine, Department of Anesthesiology and Reanimation, Faculty of Medicine, Mersin University, 33100 Mersin, Turkey; ^2^Division of Pain Medicine, Department of Anesthesiology and Reanimation, Bursa City Hospital, 16100 Bursa, Turkey

**Keywords:** Trigeminal neuralgia, Conventional radiofrequency, Peripheral nerve, Pulsed radiofrequency, Patient satisfaction, Pain relief

## Abstract

**Background**: Trigeminal neuralgia (TN) is a debilitating neuropathic pain condition with profound quality of life impact. This study aims to compare the clinical outcomes of Gasserian ganglion conventional radiofrequency (CRF) and peripheral nerve pulsed radiofrequency (PRF) in patients with TN.** Methods**: This retrospective cohort study included 74 patients with TN who underwent radiofrequency ablation (RFA) between January 2015 and June 2025 at a tertiary university pain clinic. Patients were divided into two groups: Group A (Gasserian CRF, n = 37) and Group B (Peripheral PRF, n = 37). Numerical Rating Scale (NRS) were recorded at baseline and at 1st, 3rd, and 6th months after treatment. Patient satisfaction was evaluated using a 5-point Likert scale for those with documented records. **Results**: Both groups showed significant pain relief at the 1st month compared to baseline (*p * < 0.001 for both). But, Group A showed significantly greater pain relief at the 3rd (3.54 ± 2.21 *vs.* 5.51 ± 2.91; *p* = 0.0035) and 6th months (3.19 ± 1.97 *vs.* 6.08 ± 3.06; *p* = 0.0001) than Group B. Mean satisfaction scores were significantly higher in Group A (21.76 ± 5.30) compared to Group B (14.19 ± 8.78), with a statistically significant difference (*p * < 0.001). Likert scores correlated strongly with 6-month NRS values (Spearman’s *ρ* = −0.91, *p* = 0.002). Linear regression also confirmed that lower pain scores at 6 months significantly predicted higher satisfaction (β = −2.75, *R*^2^ = 0.18, *p* = 0.003).** Conclusions**: Gasserian CRF appears more effective than peripheral PRF to ensure long-term pain relief in TN, and this may contribute to a trend toward higher patient satisfaction. Despite its invasiveness, CRF remains a valuable option for TN management. These findings support individualized procedural selection based on patient profiles and therapeutic goals. **Clinical Trial Registration**: The study was retrospectively registered on ClinicalTrials.gov (Identifier: NCT07013500).

## 1. Introduction

Trigeminal neuralgia (TN) is a chronic neuropathic pain syndrome characterized by recurrent, sudden, electric shock-like pains within the distribution of one or more trigeminal nerve branches [1]. It typically presents unilaterally and has a profound impact on patients’ daily functioning and quality of life [2]. Although pharmacological treatment with carbamazepine or oxcarbazepine remains the first-line approach, a considerable proportion of patients experience either inadequate pain relief or intolerable side effects, prompting the need for interventional procedures [3].

Radiofrequency ablation (RFA) has been widely used as a minimally invasive and safe treatment in medically refractory TN [4]. Conventional radiofrequency ablation (CRF), particularly when applied to the Gasserian ganglion via the foramen ovale, induces thermocoagulation of nociceptive fibers, resulting in prolonged pain relief [5]. However, this technique carries the risk of complications, such as corneal anesthesia, facial hypoesthesia, and masticatory muscle weakness [6].

Pulsed radiofrequency (PRF), on the other hand, is particularly favored for peripheral nerve applications due to its favorable safety profile, making it a viable option for carefully selected patients, especially those at higher risk of adverse effects [7].

Although both CRF and PRF have been utilized in the treatment of trigeminal neuralgia, comparative data on their long-term outcomes, particularly when targeting different anatomical sites such as the Gasserian ganglion versus peripheral branches, remain limited [8]. Most of the existing studies are small or examine short-term outcomes, with minimal standardization of techniques or follow-up protocols [9].

The objective of this study was, therefore, to compare the effectiveness and safety of Gasserian ganglion conventional radiofrequency ablation (CRF) and peripheral pulsed radiofrequency (PRF) in patients with classical trigeminal neuralgia. Drawing on real-world evidence from a single center over a 10-year period, this study seeks to inform clinical practice by providing further insights into optimal interventional strategies for the management of TN.

## 2. Materials and methods

### 2.1 Study design and participants

This retrospective cohort study was conducted at the Algology Department of Mersin University Faculty of Medicine. The study protocol was approved by the Clinical Research Ethics Committee of Mersin University (Approval No: 2025/606 Date: 28 May 2025). Since this was a retrospective chart-review study with anonymized data, the requirement for informed consent was waived by the Ethics Committee. Medical records of patients who underwent RFA for classical TN, between 01 January 2015 and 01 June 2025, were reviewed. Among initially identified 83 patients aged between 55 and 80 years, nine were excluded due to secondary trigeminal neuralgia, previous craniofacial surgery, or missing follow-up data. Consequently, 74 patients who met all inclusion criteria were included in the final analysis (Fig. 1). The study was registered on ClinicalTrials.gov (Identifier: NCT07013500).

**Fig. 1.** Flow diagram of patient selection. TN: Trigeminal neuralgia.

Inclusion criteria were: age between 55 and 80 years; diagnosis of classical trigeminal neuralgia according to the International Classification of Headache Disorders, 3rd edition (ICHD-3); failure to respond to medical therapy for at least six months; and availability of complete clinical and procedural data with a minimum follow-up period of six months.

Exclusion criteria included secondary trigeminal neuralgia caused by structural lesions (*e.g.*, tumors, multiple sclerosis); history of neuroablative or craniofacial surgery; missing follow-up data or patient satisfaction records; contraindications to sedation; or presence of uncontrolled severe comorbidities.

### 2.2 Study groups and RFA techniques

Patients were divided into two groups based on the radiofrequency method and anatomical target:

• Group A (Gasserian CRF):

Conventional thermal radiofrequency ablation (RFA) was performed on the Gasserian ganglion under fluoroscopic guidance in the operating room. The procedure was conducted under deep sedation. Following sensory and motor stimulation to confirm accurate needle placement, lesioning was applied at 65–75 °C for 60–90 seconds.

• Group B (Peripheral PRF):

Pulsed radiofrequency (PRF) was applied to the peripheral branches of the trigeminal nerve (supraorbital, infraorbital, or mental nerves) under ultrasound guidance in the operating room. The procedure was carried out under minimal sedation. PRF was delivered at 42 °C for a single cycle lasting 4 minutes (240 seconds).

In accordance with the standard policy of our clinic, the choice between Gasserian ganglion CRF and peripheral PRF was based on clinical judgment, taking into account the affected trigeminal branch and potential risk of complications. Specifically, peripheral PRF was preferred in cases involving the V1 or V1–V2 regions to reduce the risk of corneal hypoesthesia, keratitis or motor weakness, while CRF was typically selected for V2–V3 involvement where thermal lesioning was considered both safe and effective. As this was a retrospective study, procedural decisions were made as part of routine clinical care without randomization.

Given the retrospective nature of this study, treatment allocation was not randomized. The decision between Gasserian CRF and peripheral PRF was made by the treating physician based on clinical discretion, primarily considering the distribution of trigeminal nerve involvement and the risk profile of each technique. As such, the two groups may not be fully homogeneous, which we acknowledge as an inherent limitation of real-world, non-randomized clinical research.

### 2.3 Data collection

Demographic data (age, sex), affected side and trigeminal branch (V1, V2, V3), procedural variables (RFA type, temperature, duration), and clinical outcomes were extracted from patient records. Pain intensity was assessed using the Numeric Rating Scale (NRS) at baseline and at 1, 3, and 6 months post-procedure.

Complications (*e.g.*, corneal reflex loss, masticatory weakness, hypoesthesia) and recurrence (defined as the return of trigeminal neuralgia symptoms after initial pain relief) were documented. Procedure duration and patient satisfaction were also recorded.

Patient satisfaction was assessed retrospectively using a 5-point Likert scale (Table 1), but only in cases where satisfaction scores had been routinely documented in clinical notes. The Likert scale is a quantitative tool commonly used in clinical pain research due to its simplicity and validity in capturing patients’ subjective perception of treatment effectiveness [10]. In our clinical practice, patient satisfaction is systematically recorded following interventional procedures.

**Table 1.** Five-item Likert scale used to assess patient satisfaction.

No.	Statement
1	The treatment I received significantly reduced my pain.
2	My quality of life improved after the treatment.
3	The treatment procedure was patient-friendly.
4	I am not worried about experiencing the same pain again after the treatment.
5	I would prefer to receive the same treatment again if necessary.

*Each item was rated on a 5-point Likert scale ranging from 1 (Strongly Disagree) to 5 (Strongly Agree).*

### 2.4 Outcome measures

Primary Outcome:

• Reduction in NRS score by ≥50% at 1, 3, and 6 months.

Secondary Outcomes:

• Incidence of complications related to the procedure.

• Patient satisfaction (Likert scale).

### 2.5 Statistical analysis

Data was analyzed by SPSS version 22.0 (IBM Corp., Armonk, NY, USA). Continuous variables were checked for normality with the Kolmogorov-Smirnov test and presented as mean ± standard deviation (SD) or median (min–max) accordingly. Comparisons between groups were performed by the Student’s *t*-test or Mann-Whitney U test, depending on normality. Repeated measures were analyzed by repeated measures analysis of variance (ANOVA) or Friedman test. Categorical variables were analyzed by Chi-square or Fisher’s exact test. Additionally, the relationship between patient satisfaction scores and pain scores was evaluated using Spearman’s rank correlation analysis. To further assess predictors of patient satisfaction, a simple linear regression analysis was performed, with NRS scores as the independent variable and Likert scores as the dependent variable. *p * < 0.05 was considered statistically significant.

## 3. Results

### 3.1 Demographic and clinical characteristics

The demographic information of the patients in both groups was statistically comparable. The mean age was 63.1 ± 11.3 years in Group A and 64.4 ± 12.5 years in Group B, with no statistical difference (*p* = 0.516). In terms of sex distribution, Group A consisted of 13 males and 24 females, while Group B consisted of 19 males and 18 females. The difference in sex distribution between the groups was not statistically significant (*p* = 0.241).

In Group A, 17 patients had right-sided involvement and 20 had left-sided involvement, while in Group B, 19 patients had right-sided and 18 had left-sided trigeminal neuralgia (*p* = 0.816).

While the V1 and V1–2 branches were mostly affected in Group B (peripheral PRF), Group A (Gasserian CRF) showed higher frequencies of isolated V2, V3, and combined V2–3 involvement. One patient in Group A had all three branches (V1–2–3) affected. The difference between the two groups was statistically significant (*p* = 0.016) (Table 2).

**Table 2.** Distribution of affected trigeminal nerve branches in Group A and Group B.

Affected Branch	Group A (n)	Group B (n)
V1	0	5
V2	14	12
V3	2	1
V1–2	0	6
V2–3	20	13
V1–2–3	1	0

Complication rates were low and comparable between the two groups. In Group B (n = 37), 3 patients (8.1%) experienced complications, while in Group A (n = 37), 3 patients (8.1%) were similarly affected. The most common adverse event was transient facial hypoesthesia. Notably, no cases of corneal reflex loss or masticatory weakness were observed. There was no statistically significant difference in complication rates between the groups (*p* = 1.000).

Pharmacological treatment, such as pregabalin and carbamazepine, was discontinued in 8 patients (21.6%) of Group A and in 2 patients (5.4%) of Group B since they developed sufficient relief in symptoms. Although the rate of treatment discontinuation was higher in Group A compared to Group B, the difference was not statistically significant (*p* = 0.081).

### 3.2 Pain outcomes and patient satisfaction

The severity of pain among the patients was measured by the NRS at baseline and follow-up at 1st, 3rd, and 6th months. Pain scores at baseline (NRS_0) were similar between Group A (Gasserian CRF) and Group B (Peripheral PRF) at 9.05 ± 0.91 and 9.08 ± 0.83, respectively (*p* = 0.9265). Both of the groups on the 1st month (NRS_1) experienced a notable reduction in the pain scores from the baseline, with no statistically significant difference between Group A (4.30 ± 2.44) and Group B (4.62 ± 2.65) (*p* = 0.5761). But in the 3rd month (NRS_3), Group A reported lower pain scores (3.54 ± 2.21) compared to Group B (5.51 ± 2.91), and this was statistically significant (*p* = 0.0035). The difference was more pronounced at the 6th month (NRS_6), where Group A recorded much lower pain (3.19 ± 1.97) than Group B (6.08 ± 3.06) (*p* = 0.0001) (Table 3).

**Table 3.** Comparison of pain scores between Group A (Gasserian CRF) and Group B (peripheral PRF) over time.

	Group A (Mean ± SD)	Group B (Mean ± SD)	*p*-value
NRS_0	9.05 ± 0.91	9.08 ± 0.83	0.9265
NRS_1	4.30 ± 2.44	4.62 ± 2.65	0.5761
NRS_3	3.54 ± 2.21	5.51 ± 2.91	0.0035
NRS_6	3.19 ± 1.97	6.08 ± 3.06	0.0001

*NRS: Numerical Rating Scale; SD: standard deviation.*

The mean Likert score was significantly higher for Group A (21.76 ± 5.30) compared to Group B (14.19 ± 8.78) and indicated higher patient satisfaction among the group treated with Gasserian ganglion conventional radiofrequency ablation. The differences were statistically significant (*p* < 0.001).

In addition to the global satisfaction score, a detailed item-based analysis of the 5-point Likert scale was performed to better understand patients’ subjective treatment experiences. Patients in the CRF group reported higher scores in pain relief and quality of life improvement, whereas the PRF group scored higher in terms of perceived procedural comfort and patient-friendliness. Both groups reported similarly high willingness to undergo the same treatment again if needed. As noted in the methods section, patient satisfaction data were analyzed only for those with complete records; four patients in Group A and three patients in Group B had missing satisfaction scores, and were therefore excluded from the statistical analysis. These findings are summarized in Table 4.

**Table 4.** Distribution of individual Likert scale items in Group A (Gasserian CRF) and Group B (peripheral PRF).

Likert Item	Group A: CRF (n = 33)	Group B: PRF (n = 34)	*p*-value
1. The treatment I received significantly reduced my pain.	4.4 ± 0.6	3.6 ± 0.7	<0.001
2. My quality of life improved after the treatment.	4.2 ± 0.7	3.7 ± 0.8	0.010
3. The treatment procedure was patient-friendly.	3.6 ± 0.8	4.5 ± 0.5	<0.001
4. I am not worried about experiencing the same pain again.	4.1 ± 0.7	3.5 ± 0.8	<0.001
5. I would prefer to receive the same treatment again if necessary.	4.2 ± 0.6	4.1 ± 0.5	0.460

*CRF: conventional radiofrequency; PRF: pulsed radiofrequency.*

### 3.3 Correlation and regression analysis between pain scores and patient satisfaction

Spearman correlation test revealed there was a highly significant negative correlation between patient satisfaction (Likert scale) and pain scores at 3 months (*ρ* = −0.81) and 6 months (*ρ* = −0.91). This indicates that lower intensity of pain on follow-up was associated with higher levels of satisfaction. The correlation matrix showing the relation between the scores on NRS and satisfaction levels is presented in Fig. 2.

**Fig. 2.** Heatmap of Spearman’s correlation coefficients between NRS values and Likert satisfaction scores at four time points: baseline (NRS_0), Month 1 (NRS_1), Month 3 (NRS_3), and Month 6 (NRS_6). Warmer colors indicate more negative correlations. NRS: Numerical Rating Scale.

To explore this association in greater depth, a multivariate linear regression model was constructed, with the Likert satisfaction score as the dependent variable and NRS pain scores at baseline, and at 1-, 3-, and 6-month follow-ups as independent variables. Among these, 6th month NRS was the most significant negative predictor (β = −2.75, 95% CI (confidence interval): −3.37 to −2.13), and 3rd month NRS (β = −1.28, 95% CI: −2.08 to −0.48). Baseline NRS scores were significantly positively correlated (β = 1.91, 95% CI: 1.46 to 2.36). Fig. 3 presents the output from the multivariable linear regression model of patient satisfaction against the scores on NRS across different time points.

**Fig. 3.** Regression coefficients for predicting Likert score based on NRS values. NRS: Numerical Rating Scale.

## 4. Discussion

This real-world study compares two widely used interventional approaches for the treatment of trigeminal neuralgia: conventional radiofrequency (CRF) applied to the Gasserian ganglion and pulsed radiofrequency (PRF) applied to the peripheral branches. Our findings demonstrate that Gasserian CRF provides significantly longer-lasting pain relief and higher patient satisfaction compared to peripheral PRF. These results support the hypothesis that thermal lesioning of the Gasserian ganglion offers more durable analgesia without an increased risk of complications. To our knowledge, this is among the first direct comparisons of these two modalities utilizing mid-term follow-up and standardized outcome measures, including both pain intensity scores and patient satisfaction assessed through a validated Likert scale. These findings have the potential to inform clinical decision-making in interventional pain management by highlighting the relative efficacy and patient-centered benefits of each approach.

Previous studies have shown mixed findings regarding the relative efficacy of peripheral PRF and Gasserian CRF in trigeminal neuralgia [11, 12]. Tanyel *et al*. [13] reported that peripheral PRF may provide pain relief with fewer sensory side effects, but questioned long-term efficacy. Gasserian ganglion CRF, however, has demonstrated potent analgesia reliably but theoretically with increased risk of side effects [7]. In our study, the superior outcomes observed in the CRF group, particularly at the 3- and 6-month follow-ups, may be attributed to the more pronounced and sustained neuromodulatory effects resulting from the stimulation of the Gasserian ganglion, which houses the cell bodies of primary sensory neurons. Targeting this central anatomical site may allow for broader and longer-lasting modulation of pain transmission pathways compared to PRF applied to the distal peripheral branches [14].

Consistent with our statistical findings, the mean satisfaction scores on the 5-point Likert scale were significantly higher in the Gasserian CRF group compared to the peripheral PRF group (*p * < 0.001), suggesting a clear patient preference for the CRF technique. The multifactorial nature of determination of patient satisfaction by factors not only encompassing analgesic effectiveness but also expectations, perceived invasiveness, and prior experience, can explain variability in the results [15]. For instance, while CRF appears superior in providing long-term pain relief, its more invasive nature and potential for transient sensory disturbances may have led to lower subjective satisfaction scores for some patients [16]. Conversely, the minimally invasive nature of peripheral PRF might have resulted in relatively higher satisfaction despite its less durable analgesic effects. Furthermore, less invasive procedures are often favored by patients due to shorter hospital stays and faster recovery, factors that may contribute to increased satisfaction [17]. Therefore, integrating both objective and subjective outcome measures allows for a more comprehensive evaluation of interventional pain therapies.

Additionally, to better understand the relationship between pain intensity and patient satisfaction, Spearman correlation analysis and linear regression were conducted between follow-up NRS scores and Likert satisfaction scores. A significant negative correlation was observed, indicating that lower pain scores were associated with higher levels of satisfaction. These findings highlight the central role of effective pain control in determining patient-reported satisfaction following interventional treatments.

Our research revealed a statistically significant difference in the distribution pattern of affected trigeminal nerve branches between Group A and Group B. The group with peripheral PRF (Group B) had a higher rate of V1 and V1–2 involvement, while the Gasserian CRF group (Group A) was more commonly associated with V2, V3, and V2–3 involvement. Notably, the single patient with all three branches (V1–2–3) involved was in the Gasserian CRF group. This kind of distribution most likely covers the procedural range of each technique [18]. This observation is also consistent with previous literature recommending Gasserian interventions in cases involving the V3 branch or more extensive trigeminal nerve involvement [19].

In addition to therapeutic efficacy, technical feasibility, procedural ease, and safety are pragmatic considerations in the selection of a radiofrequency modality for trigeminal neuralgia [20]. CRF for the Gasserian ganglion is usually performed under deeper sedation or general anesthesia, given the technical complexity and invasiveness of foramen ovale cannulation [21]. This may limit its application to high-risk patients [22]. By contrast, PRF can be an option with the choice of being performed in a superficial anatomical plane, as a technically simpler and possibly safer procedure [13]. However, according to our findings, this procedural simplicity may come at the expense of reduced long-term efficacy, as reflected in the lower pain scores at the 3rd and 6th month follow-ups in the peripheral PRF group. Therefore, while PRF may be an appropriate choice for patients with contraindications to more invasive techniques, CRF should be considered the preferred option when sustained pain relief is the primary treatment goal.

In previous comparative clinical trials with trigeminal neuralgia, relative efficacy between Gasserian CRF and peripheral PRF has been controversial [23]. Yildiz *et al*. [24] documented that similar short‑term efficacy of both treatments on pain relief and reduction of medication, without differences in safety profiles. Several studies, however, have documented PRF to produce significantly worse long‑term pain relief compared with CRF [25, 26]. Consistent with prior literature, our findings confirm the superiority of Gasserian CRF in providing sustained pain relief at 3- and 6-month follow-ups, reinforcing its characterization as a technique with longer-lasting effects [27].

Thus, this study contributes to the growing body of literature by providing a direct comparison between CRF and peripheral PRF with mid-term follow-up, utilizing both objective (NRS) and subjective (Likert scale) patient-reported outcomes. The integration of these complementary outcome measures enhances the external validity of our findings and supports their applicability in guiding clinical decision-making for optimal radiofrequency strategies in trigeminal neuralgia.

## 5. Limitations

First, since it was retrospective in nature, data collection depended on existing clinical records and therefore could be a potential source of bias or lack of completeness of certain parameters, particularly in subjective outcomes such as patient satisfaction. Although the Likert scores were analyzed only in patients with systematically recorded satisfaction data, this subgroup analysis may still be susceptible to variation in documentation. Second, the sample size, while sufficient for the comparison of primary outcomes, may have had compromised power for subtle effect detection in regression or subgroup analyses. Third, the two intervention types—Gasserian CRF and peripheral PRF—also differ in procedural setting and sedation levels, as well as anatomical target and energy modality, which could influence patient comfort or perceived benefit. One limitation of the present study is the lack of detailed documentation regarding the trigeminal branch-specific distribution of complications, which prevented a more granular analysis. The retrospective chart review did not include standardized or consistent documentation of post-procedural medication adjustments; therefore, no reliable conclusion can be drawn regarding changes in analgesic medication dosage following the interventions. Finally, the follow-up period was six months; thus, long-term efficacy and recurrence prevention should be interpreted with caution and prospectively confirmed.

## 6. Conclusions

In summary, this study demonstrates that conventional radiofrequency ablation (CRF) of the Gasserian ganglion provides significantly superior mid-term pain relief compared to peripheral pulsed radiofrequency (PRF) ablation in patients with trigeminal neuralgia. Patient satisfaction also favored the Gasserian CRF group, and a significant inverse association between pain severity and satisfaction was confirmed through both correlation and regression analyses. Although peripheral PRF remains a less invasive alternative, its long-term efficacy appears to be limited. Future longitudinal studies with extended follow-up periods are warranted to validate and expand upon these findings.

## References

[1] Amaechi O. Trigeminal neuralgia: rapid evidence review. American Family Physician. 2025; 111: 427–432. 40378323

[2] Villegas Díaz D, Guerrero Alvarado G, López Medina A, Gómez Clavel JF, García Muñoz A. Trigeminal neuralgia: therapeutic strategies to restore quality of life. Journal of Oral & Facial Pain and Headache. 2024; 38: 32–37. 10.22514/jofph.2024.024 PMC11810647 39800569

[3] Murugesan I, T N U, Ramalingam K. A retrospective analysis of medical management strategies for trigeminal neuralgia: an institutional review. Cureus. 2024; 16: e69258. 10.7759/cureus.69258 PMC11470840 39398659

[4] Mansano AM, Frederico TN, Valentin REB, Carmona MJC, Ashmawi HA. Percutaneous radiofrequency ablation for trigeminal neuralgia management: a randomized, double-blinded, sham-controlled clinical trial. Pain Medicine. 2023; 24: 234–243. 10.1093/pm/pnac132 36029256

[5] Feng F, Xu Q, Wu Q, Jia C, Cai Y. Radiofrequency thermocoagulation through the foramen rotundum versus the foramen ovale for V2 primary trigeminal neuralgia: a systematic review and meta-analysis. Pain Physician. 2023; 26: E627–E633. 37847916

[6] Zheng S, Yuan R, Ni J, Liu H, Yang Y, Zhang S, *et al*. Long-term recurrence-free survival and complications of percutaneous balloon compression and radiofrequency thermocoagulation of Gasserian ganglion for trigeminal neuralgia: a retrospective study of 1313 cases. Pain Practice. 2022; 22: 532–540. 10.1111/papr.13114 35460524

[7] Lee JY, Lee GH, Yi SH, Sim WS, Kim BW, Park HJ. Non-surgical treatments of trigeminal neuralgia from the perspective of a pain physician: a narrative review. Biomedicines. 2023; 11: 2315. 10.3390/biomedicines11082315 PMC10452234 37626811

[8] Zhang X, Peng L, Liu D. Radiofrequency therapies for trigeminal neuralgia: a systematic review and updated meta-analysis. Pain Physician. 2022; 25: E1327–E1337. 36608005

[9] Howard SD, Soti V. How far has radiofrequency thermocoagulation come along as a treatment procedure in treating trigeminal neuralgia patients? Cureus. 2023; 15: e40311. 10.7759/cureus.40311 PMC10259628 37313286

[10] de Boer AGEM, van Lanschot JJB, Stalmeier PFM, van Sandick JW, Hulscher JBF, de Haes JCJM, *et al*. Is a single-item visual analogue scale as valid, reliable and responsive as multi-item scales in measuring quality of life? Quality of Life Research. 2004; 13: 311–320. 10.1023/B:QURE.0000018499.64574.1f 15085903

[11] Bharti N, Sujith J, Singla N, Panda NB, Bala I. Radiofrequency thermoablation of the Gasserian ganglion versus the peripheral branches of the trigeminal nerve for treatment of trigeminal neuralgia: a randomized, control trial. Pain Physician. 2019; 22: 147–154. 30921978

[12] Ren H, Zhao C, Wang X, Shen Y, Meng L, Luo F. The efficacy and safety of the application of pulsed radiofrequency, combined with low-temperature continuous radiofrequency, to the Gasserian ganglion for the treatment of primary trigeminal neuralgia: study protocol for a prospective, open-label, parall. Pain Physician. 2021; 24: 89–97. 33400432

[13] Tanyel T, Bilir A, Güleç MS. Peripheral nerve pulsed radiofrequency for trigeminal neuralgia treatment: is it an effective method? The Journal of the Turkish Society of Algology. 2023; 35: 96–102. 10.14744/agri.2022.93609 37052166

[14] Chen Q, Yi DI, Perez JNJ, Liu M, Chang SD, Barad MJ, *et al*. The molecular basis and pathophysiology of trigeminal neuralgia. International Journal of Molecular Sciences. 2022; 23: 3604. 10.3390/ijms23073604 PMC8998776 35408959

[15] Gavurova B, Dvorsky J, Popesko B. Patient satisfaction determinants of inpatient healthcare. International Journal of Environmental Research and Public Health. 2021; 18: 11337. 10.3390/ijerph182111337 PMC8582779 34769856

[16] Kanpolat Y, Savas A, Bekar A, Berk C. Percutaneous controlled radiofrequency trigeminal rhizotomy for the treatment of idiopathic trigeminal neuralgia: 25-year experience with 1600 patients. Neurosurgery. 2001; 48: 524–532; discussion 32–34. 10.1097/00006123-200103000-00013 11270542

[17] Quiring K, Lorio MP, León JFR, de Carvalho PST, Fiorelli RKA, Lewandrowski KU. Patient perceptions of paramedian minimally invasive spine skin incisions. Journal of Personalized Medicine. 2023; 13: 878. 10.3390/jpm13060878 PMC10305384 37373867

[18] Bangash TH. Trigeminal neuralgia: frequency of occurrence in different nerve branches. Anesthesiology and Pain Medicine. 2011; 1: 70–72. 10.5812/kowsar.22287523.2164 PMC4335746 25729659

[19] Eshraghi Y, Guirguis M, Vitug SJ. Interventional treatment options for trigeminal neuralgia. In Turker H, Garcia Benavides L, Ramos Gallardo G, Méndez-del Villar M (eds.) Peripheral Nerve Disorders and Treatment (pp. 1–19). IntechOpen: Rijeka. 2019.

[20] Elawamy A, Abdalla EEM, Shehata GA. Effects of pulsed versus conventional versus combined radiofrequency for the treatment of trigeminal neuralgia: a prospective study. Pain Physician. 2017; 20: E873–E881. 28934792

[21] Chang KW, Jung HH, Chang JW. Percutaneous procedures for trigeminal neuralgia. Journal of Korean Neurosurgical Society. 2022; 65: 622–632. 10.3340/jkns.2022.0074 PMC9452389 35678088

[22] Hong T, Ding Y, Yao P. Long-term efficacy and complications of radiofrequency thermocoagulation at different temperatures for the treatment of trigeminal neuralgia. Biochemistry Research International. 2020; 2020: 3854284. 10.1155/2020/3854284 PMC7077036 32211206

[23] Eskandar E, Kumar H, Boini A, Velasquez Botero F, El Hunjul GN, Nieto Salazar MA, *et al*. The role of radiofrequency ablation in the treatment of trigeminal neuralgia: a narrative review. Cureus. 2023; 15: e36193. 10.7759/cureus.36193 PMC10104592 37065382

[24] Yildiz G, Akkaya OT. A comparison between the efficacy of trigeminal ganglion radiofrequency thermocoagulation and ultrasound-guided maxillary-mandibular nerve pulsed radiofrequency in the treatment of trigeminal neuralgia: a randomized clinical trial. Cureus. 2024; 16: e61565. 10.7759/cureus.61565 PMC11220894 38962582

[25] Agarwal A, Rastogi S, Bansal M, Kumar S, Malviya D, Thacker AK. Radiofrequency treatment of idiopathic trigeminal neuralgia (conventional *vs.* pulsed): a prospective randomized control study. Anesthesia Essays and Researches. 2021; 15: 14–19. 10.4103/aer.aer_56_21 PMC8462427 34667342

[26] Erdine S, Ozyalcin NS, Cimen A, Celik M, Talu GK, Disci R. Comparison of pulsed radiofrequency with conventional radiofrequency in the treatment of idiopathic trigeminal neuralgia. European Journal of Pain. 2007; 11: 309–313. 10.1016/j.ejpain.2006.04.001 16762570

[27] Wang Y, Jia Y, Wang Z, Feng G, Ma Y, Fan Z, *et al*. Efficacy and safety of high-voltage pulsed radiofrequency versus standard-voltage pulsed radiofrequency for patients with neuropathic pain: a literature review and meta-analysis. Journal of Pain Research. 2024; 17: 851–863. 10.2147/JPR.S439909 PMC10922952 38464903

